# Salvage Aneurysmorrhaphy as an Adaptable and Still Pertinent Technique in the Management of Challenging True Aneurysms of Arteriovenous Fistulas: A Case Series of Different Variations, With Illustrative Surgical Pictures

**DOI:** 10.1016/j.ejvsvf.2024.05.002

**Published:** 2024-05-10

**Authors:** Homa Pourriyahi, Homayoun Pourriahi, Hossein Najd Sepas

**Affiliations:** a School of Medicine, Iran University of Medical Sciences, Tehran, Iran; b Department of Vascular Surgery, Rasool-e-Akram Hospital, Iran University of Medical Sciences, Tehran, Iran

**Keywords:** Aneurysm, Aneurysmoplasty, Aneurysmorrhaphy, Arteriovenous fistula, Megafistula, Partial aneurysmectomy

## Abstract

**Introduction:**

Aneurysmorrhaphy, described as reduction aneurysmoplasty, partial aneurysmectomy, or vessel wall recalibration, can be considered a suitable surgical plan for true aneurysms of arteriovenous fistulas (AVFs), allowing for a dynamic approach to reconstruction of aneurysmal AVFs of different severities, ensuring salvage of the native access.

**Report:**

Six challenging cases of AVF aneurysms are presented, some with extremely dilated and tortuous megafistulas, for which three surgical technique variations were performed. The patients had a mean age of 59.2 years, 50% were female, with brachiocephalic (*n* = 5, 83.3%) or brachiobasilic (*n* = 1, 16.7%) AVFs. The fistulas were created an average of 4.67 years previously, and the aneurysms had an average maximum diameter of 37.5 mm (range 25–60 mm). Surgical indications were rupture risk, thrombosis, or outflow stenosis compromising haemodialysis, infections, and concerns for quality of life (affected by post-puncture bleedings, disfiguring aesthetics, pain, and discomfort). The surgical techniques were simple aneurysmorrhaphy (*n* = 3, 50%), aneurysmorrhaphy with partial excision of aneurysmal segment with end to end anastomosis of venous ends (*n* = 2, 33.3%), and aneurysmorrhaphy with establishment of new venous outflow (*n* = 1, 16.7%). All AVFs were patent post-operatively and at follow up (mean 5.6 months, median one month). Haemodialysis was resumed through the AVFs at a mean of 2.17 weeks post-operatively, with placement of an alternative route for haemodialysis in the meantime. No patients experienced post-operative complications.

**Discussion:**

Experience with the more challenging cases shows that aneurysmorrhaphy can still be considered an acceptable, flexible, and pertinent method for salvage of megafistulas, giving the surgeon the much needed versatility to adapt to anatomical and pathological variations, with high patency rates and minimal complications, especially when other treatment options are not possible in complicated cases. AVF salvage through aneurysmorrhaphy allows for a dynamic approach to the reconstruction of severely tortuous, dilated veins, ensuring patency of the native AVF.

## Highlights

• Experience with the more challenging cases shows that aneurysmorrhaphy can still be considered an acceptable, flexible and pertinent method for salvage of severely aneurysmatic arteriovenous fistulas (AVFs), despite it not currently being a technique in the spotlight.
• Adapting to anatomical and pathological variations is the heart of vascular surgery and is essential to ensuring patency of the native AVF with minimum complications, especially when the extent of the pathology does not allow for other treatment options.
• Megafistulas are treatable with good outcomes, even in complicated patients with comorbidities, given the proper surgical care.

## Introduction

Native arteriovenous fistulas (AVFs) are the access of choice for haemodialysis, with low risks of infection and high patency rates compared with grafts.^1^ Nevertheless, complications do occur in one third of fistulas, e.g., thrombosis, stenosis, aneurysmal degeneration, and infection.^1^ To define an aneurysm for native access, it should be noted that AVFs are sited to cause an abnormal dilatation of the respective vessel, and although different definitions have been proposed for an aneurysmal AVF, a diameter of 18 mm and above ^2^^,^^3^ or three times the acceptable maturation diameter^4^^,^^5^ are commonly agreed upon. In more severe cases, where the vessel becomes severely tortuous and dilated, with flow rates of above 2 000 mL/min, the AVF is referred to as a megafistula.^6^^,^^7^

Management of aneurysmal AVFs is highly individualised, and as aneurysms develop different morphologies, leading to variable performance deficits, a uniform conservative or surgical plan cannot be described for all.^8^ Indications for surgery are mainly due to complications such as rupture risk, thrombosis, or outflow stenosis compromising haemodialysis, infections, and, moving on from life threatening implications, concerns for a patient's quality of life and daily performance, which can be severely diminished by multiple post-puncture bleedings, disfiguring aesthetics, pain, and discomfort, are also taken into account.^8^

When indicated, the first surgical choice should be a plan to salvage the native access, ideally avoiding prostheses, new anastomoses, or ligation.^6^^,^^8^ Aneurysmorrhaphy, described as reduction aneurysmoplasty,^1^ partial aneurysmectomy and repair,^9^ or vessel wall recalibration,^4^ can be considered a suitable surgical plan, especially for megafistulas, that focuses on native reconstructive salvage of the access, while allowing for variability in surgical technique to suit the situation at hand. Six versatile cases are presented in which aneurysmorrhaphy was used to successfully salvage the aneurysmal AVFs. This case series has been reported in line with the PROCCESS guideline.^10^

### Ethics approval and consent to participate

This was a retrospective case series of six challenging cases, for which a chart review was carried out, along with the use of previously taken surgical images, for which the patients provided written consent pre-operatively.

Patients whose information is shared in this article, without mention of their identity or personal data, agreed to the use of their respective courses of treatment and surgical images in this study and provided written informed consent for the sharing of their medical history and surgical images in a journal article post-operatively and or on follow up visits.

The course of treatment or surgical management of the patients was not altered in any way relating to this study. This study was conducted in line with our ethics department policy for case reports.

Data associated with this work is available from the corresponding author upon reasonable request.

## Case reports and surgical technique

The most important pearl in the surgical care of aneurysmal AVFs is making sure pre-operatively that no stenoses or occlusions are present proximal to the fistula, e.g., in the cephalic or subclavian veins. This is established through venography, which is the standard of care, and directs the course of surgery, as there might be a need for thrombectomy or establishing new venous outflow. Under local anaesthetic, a semilunar incision is made on the skin over the aneurysm. Care is taken in separating the thin skin over the aneurysm from the vessel wall. The aneurysmal vein is dissected throughout its length and released from surrounding tissues.

After systemic heparinisation and clamping of the vein on proximal and distal non-aneurysmal segments with vascular clamps, a longitudinal incision is made on the vein with a scalpel and the thrombi and fibrotic tissues in the lumen are removed. The dilated vessel is calibrated to the diameter of a 28 Fr chest tube by partial resection of the redundant aneurysmal wall. The defect in the venous wall is then closed with a continuous locking 4-0 Prolene suture. If the calibrated vessel is longer than desirable, meaning it lies tortuously in the new vessel bed, a portion of the vein is transected and excised. The cut ends of the continuous locking suture are secured to prevent loosening of the previous stitches. Venous continuity will then be established with an end to end anastomosis. Lastly, the clamps are removed and with the establishment of haemostasis, the overlying skin flap is closed with a simple interrupted 4-0 nylon suture. The details of cases are summarised in Table 1.

**Table 1:** Case characteristics.

Patient number	Age, sex	Underlying diseases	AVF site, age	Aneurysm size	Surgical indication	Surgical technique	Temporary catheter and durations	Outcomes and Complications
Case 1	65 y, F	DM, IHD, and ESRD	Left BB, 1 y	L: 3 dilations, 20–40 mm each; D: 25 each	Pulsatile mass, recalibration before superficialisation of already in use AVF	Simple aneurysmorrhaphy (*n* = 3, 50%)	Right jugular non-tunnelled CVC for 2 wk	Patent newly superficialised AVF used for HD after 2 wk/none at 4 wk
Case 2	35 y, F	DM, ESRD	Left BC, 3 y	L: 2 dilations, 40, 50 mm; D: 25 mm each	Compromised AVF outflow due to thrombosis		Right jugular tunnelled CVC for 2 wk	Patent AVF used for HD after 2 wk/none at 4 wk
Case 3	67 y, M	ESRD	Right BC, 10 y	L: longer than patient's upper arm; D: 40 mm	Megafistula, Compromised AVF outflow due to thrombosis		Right jugular non-tunnelled CVC for 2 wk	Patent AVF used for HD after 2 wk/none at 4 wk
Case 4	45 y, M	ESRD	Left BC, 4 y	L: longer than patient's arm; D: 40 mm	Megafistula, thrombosis, multiple bleedings due to thinned skin	Aneurysmorrhaphy with partial excision of the aneurysmal segment with end to end anastomosis of the venous ends (*n* = 2, 33%)	Right jugular tunnelled CVC for 3 wk	Patent AVF used for HD after 3 wk/none at 6 mo
Case 5	75 y, M	ESRD	Left BC, 5 y	L: longer than patient's upper arm; D: 60 mm	Megafistula, thrombosis, multiple bleedings due to thinned skin		Right femoral non-tunnelled CVC for 2 wk	Patent AVF used for HD after 2 wk/none at 2 wk, lost to follow up thereafter
Case 6	68 y, F	HTN, ESRD	Left BC, 5 y	L: 2 dilations, 50 mm each; D: 35 mm	Compromised AVF outflow due to thrombosis and central stenosis	Aneurysmorrhaphy with establishment of a new venous outflow (*n* = 1, 17%)	Right femoral non-tunnelled CVC for 2 wk	Patent AVF used for HD after 2 wk/none at 24 mo
Total	Mean age: 59.2 y, 50% F	ESRD (*n* = 6, 100%), DM (*n* = 2, 33.3%), HTN (*n* = 1, 16.7%), IHD (*n* = 1, 16.7%)	BC (*n* = 5, 83.3%), BB (*n* = 1, 16.7%), for an average 4.67 y	Average max diameter = 37.5 mm (range 25–60 mm)	Aneurysm (*n* = 6, 100%), megafistula (*n* = 3, 50%), thrombosis (*n* = 5, 83.3%), bleeding (*n* = 2, 33.3%), pulsatile mass (*n* = 1, 16.7%), central venous stenosis (*n* = 1, 16.7%)			Patent AVFs used for HD after an average of 2.17 wk/none at follow up (mean 5.6 mo, median 1 mo)

F = female; M = male; HTN = hypertension; ESRD = end stage renal disease; DM = diabetes mellitus; IHD = ischaemic heart disease; BC = brachiocephalic; BB = brachiobasilic; L = length; D = diameter; AVF = arteriovenous fistula; HD = haemodialysis; CVC = central venous catheter.

### Simple aneurysmorrhaphy

#### Case 1

The first case was a 65 year old woman with diabetes mellitus (DM), ischaemic heart disease (IHD), and end stage renal disease (ESRD). She underwent brachiobasilic AVF placement last year, which developed three aneurysmal segments in the basilic vein during the six months that it had been used for dialysis before superficialisation. The patient presented to the clinic complaining of a pulsatile mass in her arm one year after AVF placement. A simple aneurysmorrhaphy was performed, recalibrating the aneurysmal vein. The vein had an acceptable length, but as it had been newly reconstructed a classic transposition was not performed. A pedicled subcutaneous flap was made from the patient's subcutaneous tissue on the anteromedial arm, with the reconstructed vein placed on top of the flap, and the medial side of the flap then closed under the skin with 4-0 Polydioxanone (PDA) interrupted sutures, so the vein was superficialised for better access (Supplementary Figure S1).

#### Case 2

The second case is a 35 year old woman with DM and ESRD who underwent brachiocephalic AVF placement three years previously. Aneurysmal degeneration of the AVF began one year ago, eventually leading to thrombosis and total compromise of AVF outflow for a week, before her presentation. Thrombectomy was performed, requiring an additional incision to extend the thrombectomy proximally, plus aneurysmorrhaphy with adjustment of the venous wall (Supplement Figure S2).

#### Case 3

The third case was a 67 year old man with ESRD who underwent brachiocephalic AVF placement 10 years ago. Aneurysmal degeneration of the AVF began four years ago, leading to thrombosis and partial compromise of AVF outflow before revision. Thrombectomy with simple aneurysmorrhaphy was performed (Supplementary Figure S3).

### Aneurysmorrhaphy with partial excision and re-anastomosis

#### Case 4

The fourth case was a 45 year old man with ESRD who was dialysed through a brachiocephalic AVF placed four years ago. The patient had undergone stenting of the cephalic vein in another centre due to stenosis, which led to severe aneurysmal degeneration of the venous portion of the AVF. He also reported two incidents of severe haemorrhage from the aneurysmal AVF, as the overlying skin had become very thin and easily ruptured. Pre-operative venography showed severe thrombosis of the aneurysmal cephalic vein which had caused total occlusion of venous blood flow.

**Figure 1:**
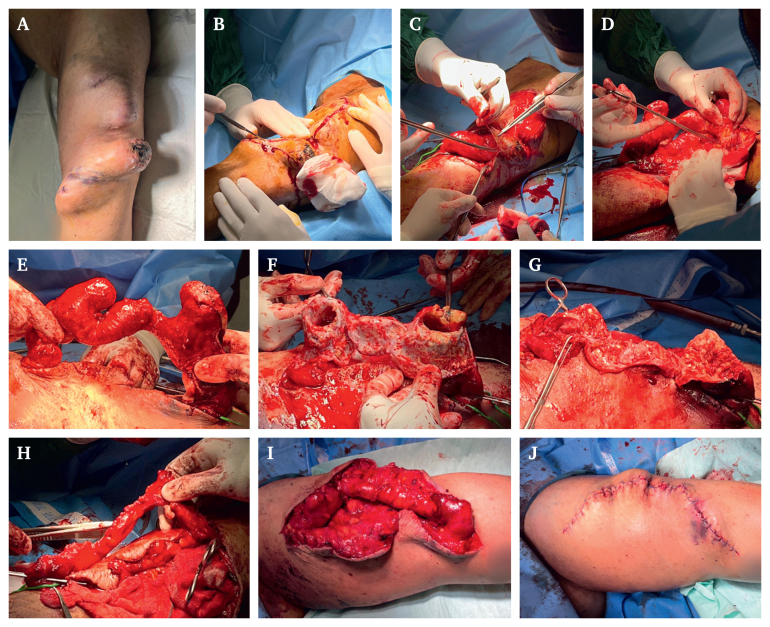
Case 4. (A) Preoperative. (B) Longitudinal and elliptical incisions. (C–E) Dissection of the aneurysmal vein from the vessel bed. (F) After removal of thrombi and fibrotic tissue from the vein. (G) After removal of the redundant vessel wall. (H) Aneurysmorrhaphy with calibration to a 28 Fr chest tube. (I) After removal of clamps and establishment of haemostasis. (J) Simple interrupted suture of the skin (patient's tattoos have been slightly blurred in images to protect their privacy).

Two incisions were made: first, a longitudinal incision over the course of the dilation, and second, an elliptical continuation of the first incision over the partially necrotic scabbed skin over the severely dilated segment of the vein. The scabbed part was not dissected and was left on the vein, where it was later removed together with the redundant venous wall. After removal of the thrombi and the previously placed stent entrapped in atheromatous plaques, the venous wall was recalibrated and the reconstructed vein was shortened to lie appropriately in the vessel bed (Fig. 1).

#### Case 5

**Figure 2:**
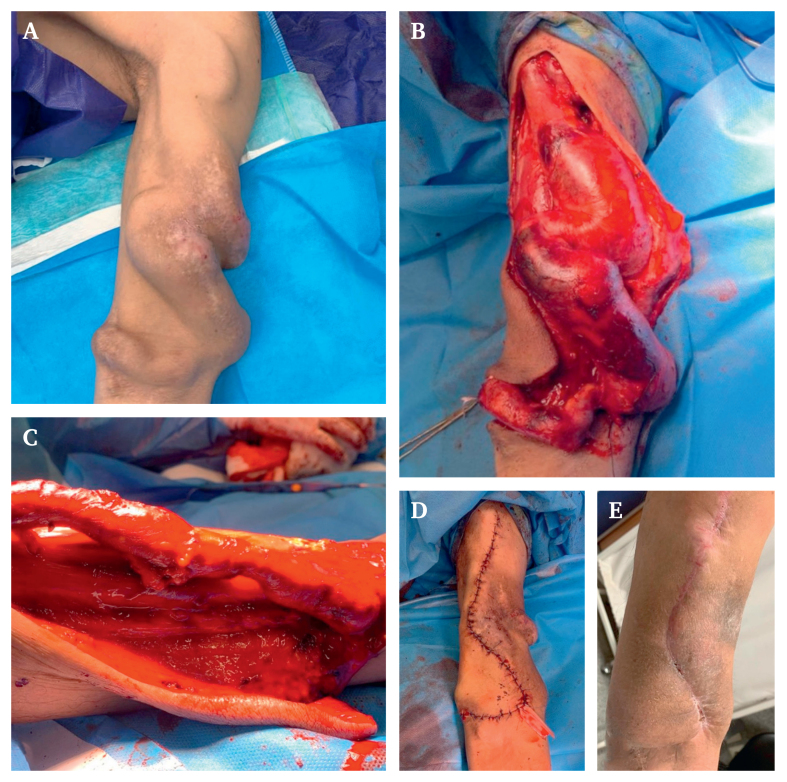
Case 5. (A) Preoperative. (B) Dissection of the aneurysmal vein. (C) Aneurysmorrhaphy with calibration to a 28 Fr chest tube. (D) Simple interrupted suture of the skin. (E) Two weeks post-operative.

The fifth case was a 75 year old man with ESRD, who underwent radiocephalic AVF placement five years ago, and presented to the clinic with a severely tortuous aneurysm of the cephalic vein, which had resulted in multiple bleedings from wounds on the stretched skin. After aneurysmorrhaphy of the cephalic vein, part of the vessel was resected and re-anastomosed to achieve a desirable venous length (Fig. 2).

### Aneurysmorrhaphy with new venous outflow

#### Case 6

The last case was a 68 year old female with hypertension (HTN) and ESRD who had undergone haemodialysis three times a week for the past five years. A brachiocephalic AVF was placed three months after the beginning of her dialysis. The AVF began aneurysmal degeneration two years after its placement, with progressive reduction in the AVF flow rate, which had the patient presenting for evaluation a year afterwards. Pre-operative venography showed severe stenosis in the left cephalic arch. The stenosis encompassed the cephalic arch, extending to the left cephalic–axillary vein junction.

After simple aneurysmorrhaphy of the dilated cephalic vein in the upper arm, the cephalic vein was ligated just distal to the stenotic cephalic arch; the cephalic vein was then transected distal to the stenosis and venous drainage was established by interposition of an 8 cm polytetrafluoroethylene (PTFE) jump graft in place of the cephalic arch between the distal newly cut end of the cephalic vein onto the anterolateral side of the left subclavian vein (about 3 cm medial to the cephalic axillary junction) through the clavipectoral fascia. Care was taken in the dissection around the thoraco-acromial artery and the lateral pectoral nerve. Unfortunately, no images were taken during surgery.

## Discussion

Six cases have been presented with severe multiple aneurysmal degeneration AVFs requiring haemodialysis access. The aneurysms were successfully treated by different aneurysmorrhaphy techniques.

Several different techniques in aneurysmorrhaphy have been proposed, namely simple, partial excision with end to end anastomosis, establishment of new venous drainage, stapler assisted, reinforced with an exoprosthesis, plication, and other innovative techniques. Three of these techniques were employed, with modifications and variations, even for the same techniques, to salvage the respective fistulas.

As stenoses and aneurysms often coexist, with a reported 13–100% incidence of stenoses compromising outflow in aneurysmal AV accesses, venography prior to non-emergency surgery has been recommended.^4^ Venography was also performed prior to all operations which rightfully so, guided plans for establishment of adequate venous outflow.

For continuation of dialysis while surgical wounds heal, temporary tunnelled catheters were placed. In addition, there were studies of immediate haemodialysis after corrective surgery, either by placing the suture line on the reconstructed vessel posteriorly,^6^ so it was covered by the vessel bed and away from puncture sites, or by staging the aneurysmorrhaphy for multiple dilatations, so dialysis could be resumed through non-operated segments.^9^ Patients received jugular non-tunnelled central venous catheters (CVCs) (*n* = 2, 33%), right femoral non-tunnelled CVCs (*n* = 2, 33%), and right jugular tunnelled CVCs (*n* = 2, 33%). Choosing between tunnelled and non-tunnelled was a concern, as the duration of the healing process was uncertain, given the demographics and underlying morbidities of the patients. Living with a non-tunnelled catheter was a challenge for cases 2 and 4 as they had corporate jobs. Non-tunnelled catheters were sited for all but these two younger patients, and removed two to three weeks after placement as the fistulas were fully healed by then, but a non-tunnelled CVC should suffice in these settings and should be the standard of practice.

The patients were visited every day for the first post-operative week and then every three days; the wounds were nicely healed by week 2. We waited one additional week (three weeks total) for one of the patients (case 4) to re-puncture the AVF as the tissue removal was truly extensive and the patient had undergone several re-operations before visiting the clinic (see Fig. 1 for the extent of the patient's aneurysms), and the skin was very thin due to expansion.

Complications are mostly minimal in the published literature: mainly infections or recurrences, or rare thromboses.^6^ Although complications had been expected as the patients were mostly older, some had diabetes, and extensive tissue removal was performed, no complications were observed during follow up.

In conclusion, aneurysmorrhaphy can be considered an acceptable, applicable, and flexible method for salvage of aneurysmal AVFs, giving the surgeon the much needed versatility to adapt to anatomical and pathological variations, with high patency rates and minimal complications, especially when other treatment options are not suitable to perform on a complicated megafistula. AVF salvage through aneurysmorrhaphy allows for dynamic reconstruction of severely tortuous and dilated veins, ensuring patency of the native AVF.

## Funding

None.

## Conflict of interest

None.

## Appendix A Supplementary data

All Supplementary Figures
