# Supplementary material for: Salvage Aneurysmorrhaphy as an Adaptable and Still Pertinent Technique in the Management of Challenging True Aneurysms of Arteriovenous Fistulas: A Case Series of Different Variations, With Illustrative Surgical Pictures
Source: EJVES Vasc Forum. 2024 May 10;61:126–31. doi: 10.1016/j.ejvsvf.2024.05.002 (PMC11177082; doi:10.1016/j.ejvsvf.2024.05.002)
Supplement: Supplementary Figure S1 [file mmc2.pdf]

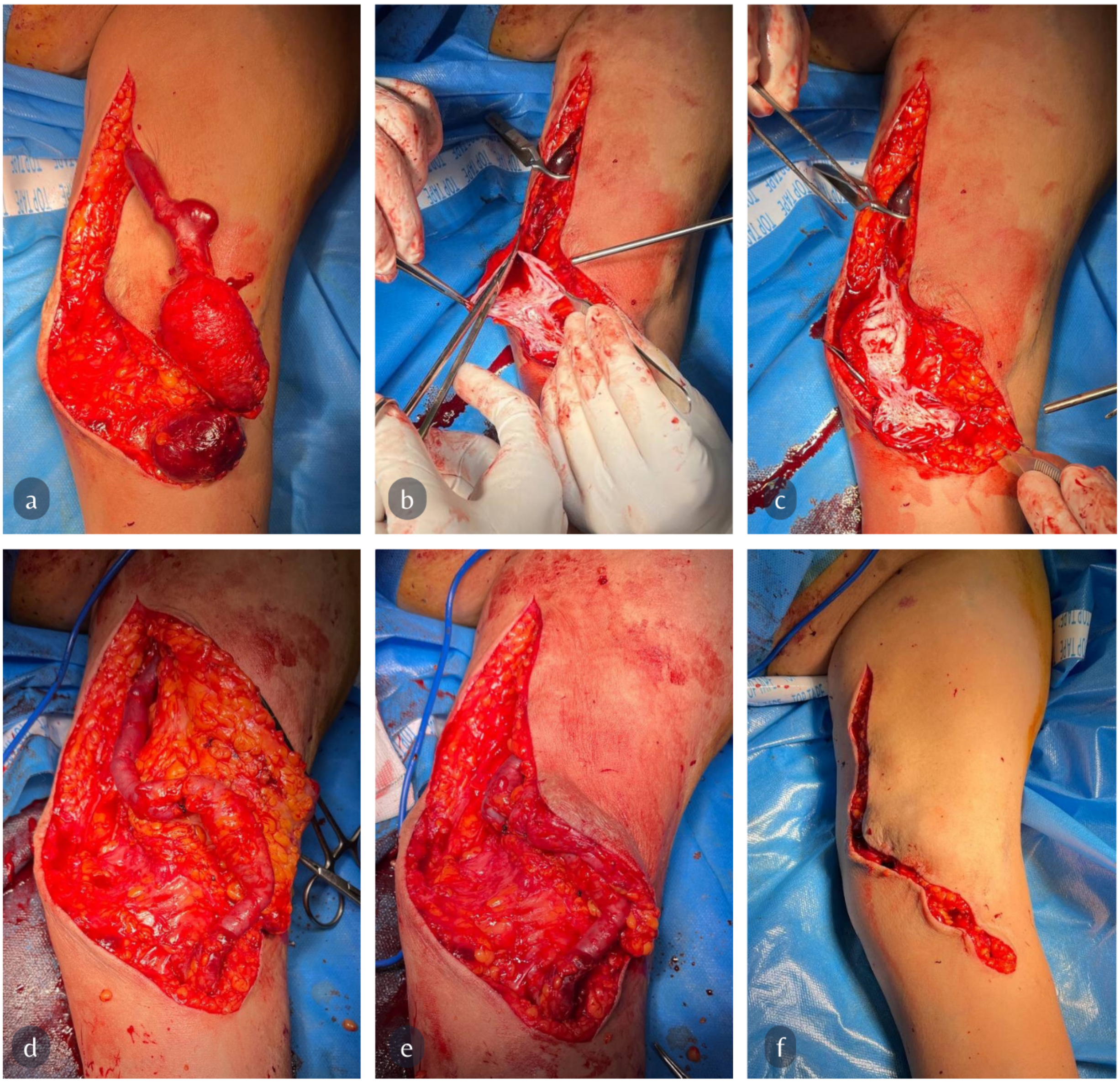

Supplement Figure 1. Case 1: a. dissection of the aneurysmal vein from the vessel bed, b. longitudinal incision on the aneurysmal vessel, c. after removal of the redundant vessel wall, d. after aneurysmorrhaphy and establishment of hemostasis, e. placement of the repaired vein over a subcutaneous flap, f. simple interrupted closure of the subcutaneous tissue. Note: The patient had not undergone superficialization/elevation yet, but had been using the fistula for dialysis through some puncture areas which had caused stenoses and aneurysms. We dissected the basilic vein which had multiple aneurysmal sections, performed the aneurysmorrhaphy, and as you can see in Supplement Figure 1, planes d, e and f, we made a pedicled subcutaneous tissue flap from the tissue under the skin on the patient's anteromedial arm, placed the reconstructed vein on top of the flap, then closed the medial side of the flap under the skin with 4-0 Polydioxanone (PDA) interrupted sutures. We then closed the skin with interrupted nylon sutures.
