# Supplementary material for: Salvage Aneurysmorrhaphy as an Adaptable and Still Pertinent Technique in the Management of Challenging True Aneurysms of Arteriovenous Fistulas: A Case Series of Different Variations, With Illustrative Surgical Pictures
Source: EJVES Vasc Forum. 2024 May 10;61:126–31. doi: 10.1016/j.ejvsvf.2024.05.002 (PMC11177082; doi:10.1016/j.ejvsvf.2024.05.002)
Supplement: Supplementary Figure S2 [file mmc3.pdf]

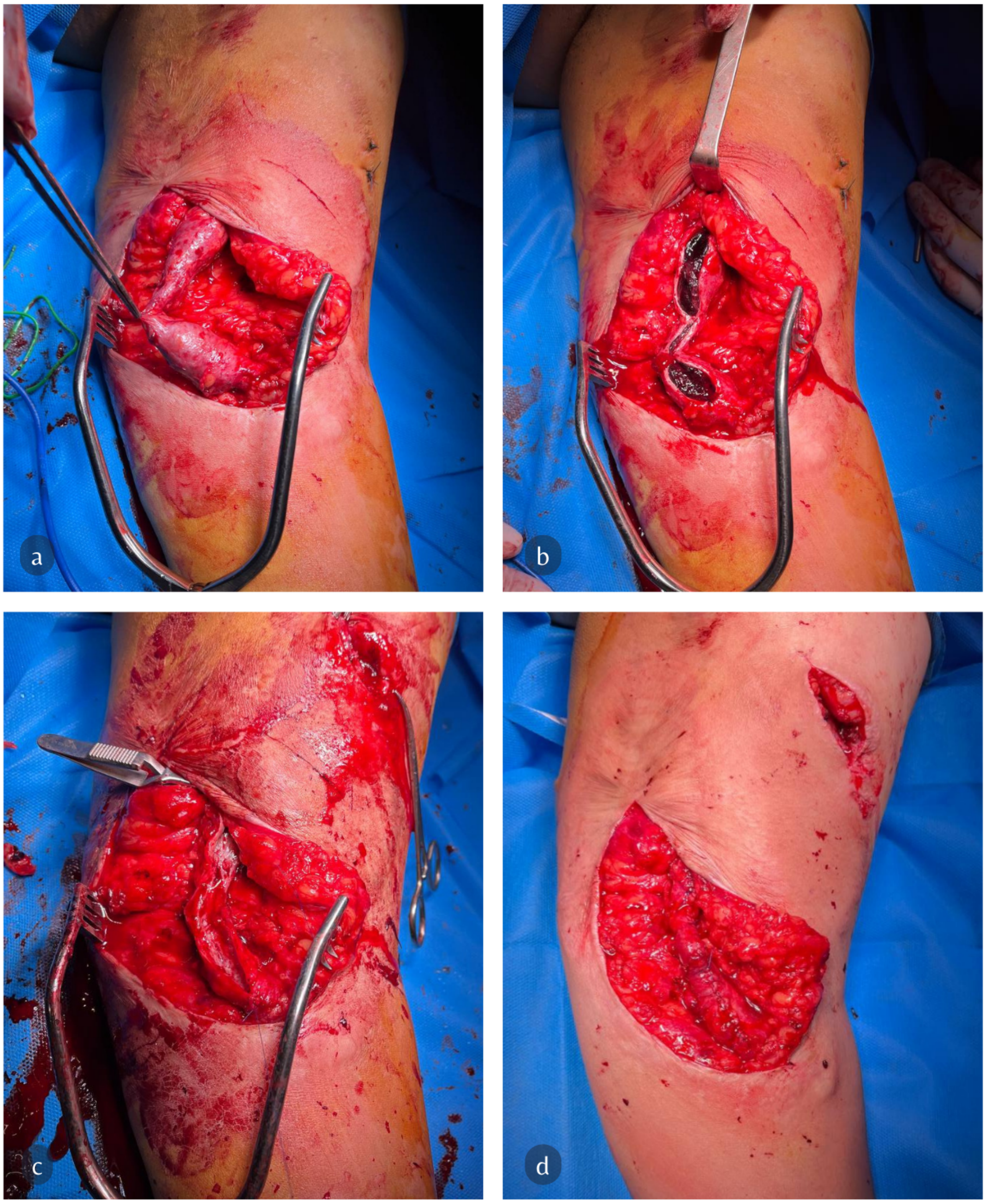

Supplement Figure 2. Case 2: a. dissection of the aneurysmal vein from the vessel bed, b. thrombi and fibrotic tissue occluding the aneurysmal fistula, c. thrombectomy (requiring another proximal incision) and removal of the redundant vessel wall, d. aneurysmorrhaphy and establishment of hemostasis.
