# Supplementary material for: Salvage Aneurysmorrhaphy as an Adaptable and Still Pertinent Technique in the Management of Challenging True Aneurysms of Arteriovenous Fistulas: A Case Series of Different Variations, With Illustrative Surgical Pictures
Source: EJVES Vasc Forum. 2024 May 10;61:126–31. doi: 10.1016/j.ejvsvf.2024.05.002 (PMC11177082; doi:10.1016/j.ejvsvf.2024.05.002)
Supplement: Supplementary Figure S3 [file mmc4.pdf]

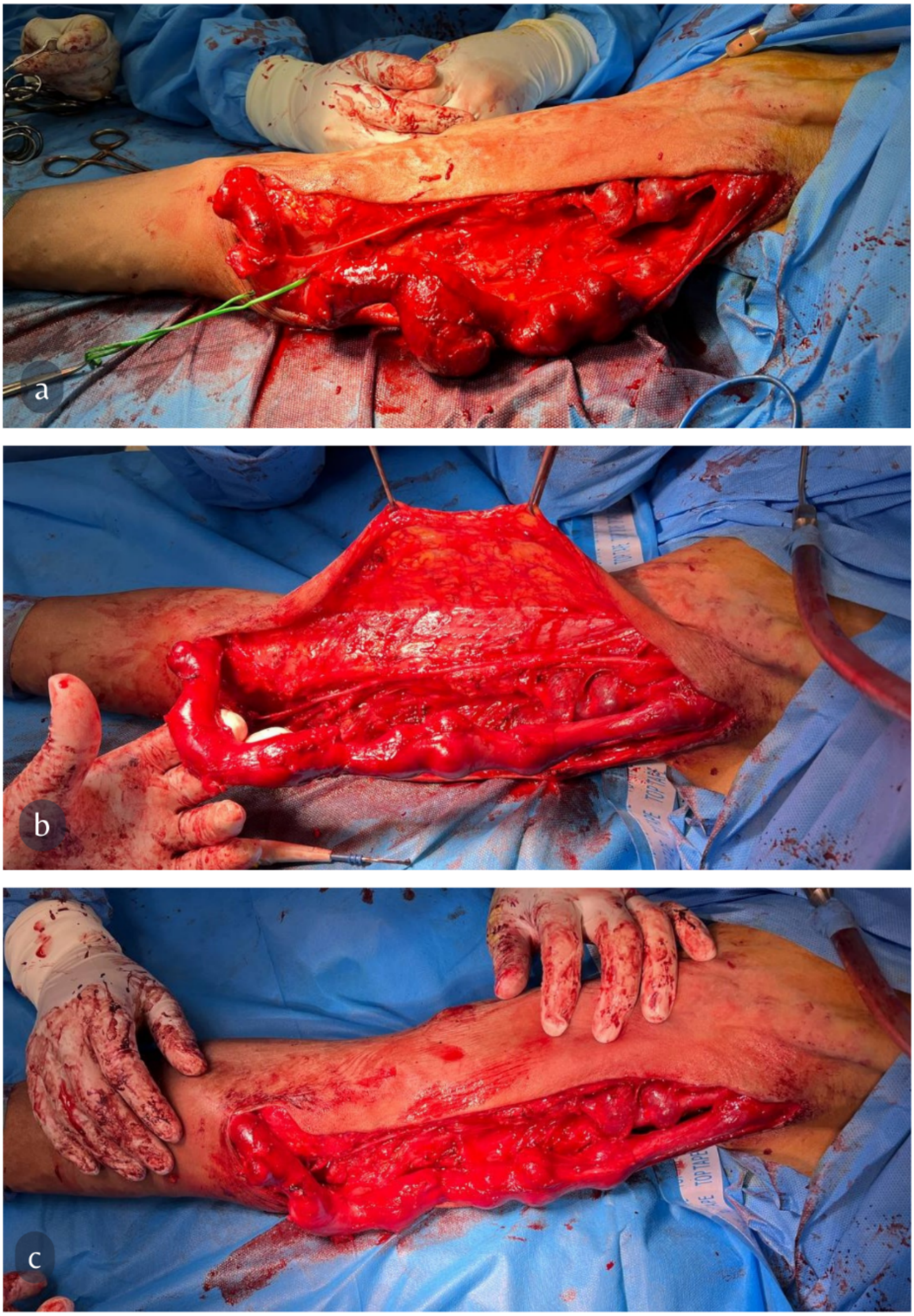

Supplement Figure 3. Case 3: a. dissection of the aneurysmal vein from the vessel bed, b,c. aneurysmorrhaphy and establishment of hemostasis.
